# Prediction of carbapenem-resistant gram-negative bacterial bloodstream infection in intensive care unit based on machine learning

**DOI:** 10.1186/s12911-024-02504-4

**Published:** 2024-05-14

**Authors:** Qiqiang Liang, Shuo Ding, Juan Chen, Xinyi Chen, Yongshan Xu, Zhijiang Xu, Man Huang

**Affiliations:** 1 General Intensive Care Unit and Key Laboratory of Multiple Organ Failure, China National Ministry of Education, Second Affiliated Hospital of Zhejiang University School of Medicine, No. 1511, Jianghong Road, Bingjiang District, Hangzhou, Zhejiang China; 2 Laboratory Chief, Key Laboratory of Multiple Organ Failure, China National Ministry of Education, Hangzhou, Zhejiang China; 3 Clinical Laboratory, Second Affiliated Hospital of Zhejiang University, Hangzhou, Zhejiang China

**Keywords:** Carbapenem-Resistant gram-negative bacteria, Bloodstream infection, Machine learning, Intensive care unit, Prediction model

## Abstract

**Background:**

Predicting whether Carbapenem-Resistant Gram-Negative Bacterial (CRGNB) cause bloodstream infection when giving advice may guide the use of antibiotics because it takes 2–5 days conventionally to return the results from doctor's order.

**Methods:**

It is a regional multi-center retrospective study in which patients with suspected bloodstream infections were divided into a positive and negative culture group. According to the positive results, patients were divided into the CRGNB group and other groups. We used the machine learning algorithm to predict whether the blood culture was positive and whether the pathogen was CRGNB once giving the order of blood culture.

**Results:**

There were 952 patients with positive blood cultures, 418 patients in the CRGNB group, 534 in the non-CRGNB group, and 1422 with negative blood cultures. Mechanical ventilation, invasive catheterization, and carbapenem use history were the main high-risk factors for CRGNB bloodstream infection. The random forest model has the best prediction ability, with AUROC being 0.86, followed by the XGBoost prediction model in bloodstream infection prediction. In the CRGNB prediction model analysis, the SVM and random forest model have higher area under the receiver operating characteristic curves, which are 0.88 and 0.87, respectively.

**Conclusions:**

The machine learning algorithm can accurately predict the occurrence of ICU-acquired bloodstream infection and identify whether CRGNB causes it once giving the order of blood culture.

**Supplementary Information:**

The online version contains supplementary material available at 10.1186/s12911-024-02504-4.

## Background

Among the infections of severe patients, the mortality of bloodstream infections is the highest [1]. Recently, with the prevalence of multiple drug-resistant bacteria (MDR) in China, bloodstream infections caused by MDR are becoming more common in critically ill patients [2]. Common clinical MDRs include carbapenem-resistant *Enterobacteriaceae* (CRE), carbapenem-resistant *Acinetobacter baumannii* (CRAB), and carbapenem-resistant *Pseudomonas aeruginosa* (CRPA), which has been on the list of priority pathogens by the World Health Organization [3]. Today, the most common and dangerous MDR in China is Carbapenem-resistant gram-negative bacilli (CRGNB). Empirical antibiotic therapy becomes very difficult once MDR must be considered due to different drug resistance mechanisms, local epidemiology, site of infection, immune status, history of antimicrobial exposure, and MDR colonization records [2].

Blood culture and susceptibility tests, the gold standard of bloodstream infection, guide the empirical antibiotic regimen to targeted use [1]. However, the blood culture needs 1–3 days from sampling to results, depending on the bacterial load in the blood, making it relatively hysteretic to diagnose bloodstream infections [4]. Additionally, this duration may lead to sepsis progression to septic shock and even death which cannot meet the demand of time-racing rescue of sepsis [5]. If there are artificial intelligence predictive tools to remind the incidence of drug-resistant bacteria bloodstream infection when doctors make the order of blood culture, it may help clinicians to determine the antibiotic regimen, which may be helpful to the control of bloodstream infection.

The use of machine learning to predict bloodstream infection of MDR provides a possible solution from another point of view, which is more meaningful for guiding the use of antibiotics. Application of machine learning in MDR-GNB is increasing, including predicting the risk of MDR-GNB infection, predicting whether known infections originate from MDR-GNB, and guiding antibiotic management and prevention and control of MDR-GNB. According to current research, predicting the occurrence of bloodstream infection is feasible [6–10]. Michael Roimiuse and his colleagues used the MIMIC and RHCC databases to predict acquired bloodstream infection in patients with suspected ICU infection using the XGBoost prediction model. The area under the receiver operating characteristic (AUROC) of two centers are 0.89 ± 0.01 and 0.92 ± 0.02, respectively [10]. Four other centers built machine-learning prediction models of bloodstream infection through their central databases, and the AUROC of models varied from 0.77 to 0.82 [6, 8, 9, 11]. No studies predict the occurrence of MDR bloodstream infection, and the guidance for the current situation of MDR bloodstream infection is limited. As the most common and virulent type of MDR, CRGNB are the focus of our study. The antibiotic regimen is determined according to the patient's state, the degree of sepsis shock, the type of bacteria prevalent in the ward once doctors suspect bloodstream infection, and our artificial intelligence can provide some advices for antibiotic use early. Normally, the most commonly used antibiotics were broad-spectrum antibiotics rather than these treat drug-resistant bacteria, including colistin, ceftazidime-avibactam, and tigecycline. This study focuses on predicting whether the pathogenic bacteria of bloodstream infection are MDR bacteria using machine learning algorithms.

## Methods

### Study characteristics

The database in our study is from the general intensive care unit (ICU) database (SHZJU-ICU) of the second affiliated hospital of the Zhejiang University School of Medicine, a large academic teaching hospital in southeast China with 3800 beds in four districts of Hangzhou. General ICU has set up four wards in three of these districts, with 26 beds, 40 beds, and 10 beds to treat critically ill patients independently. Data from the three wards were shared in 2021. A total of approximately 18000 critically ill patients were included in this database, included demographics, vital signs, clinical examination, medication orders, clinical diagnosis, and medical documents.

**Fig. 1:**
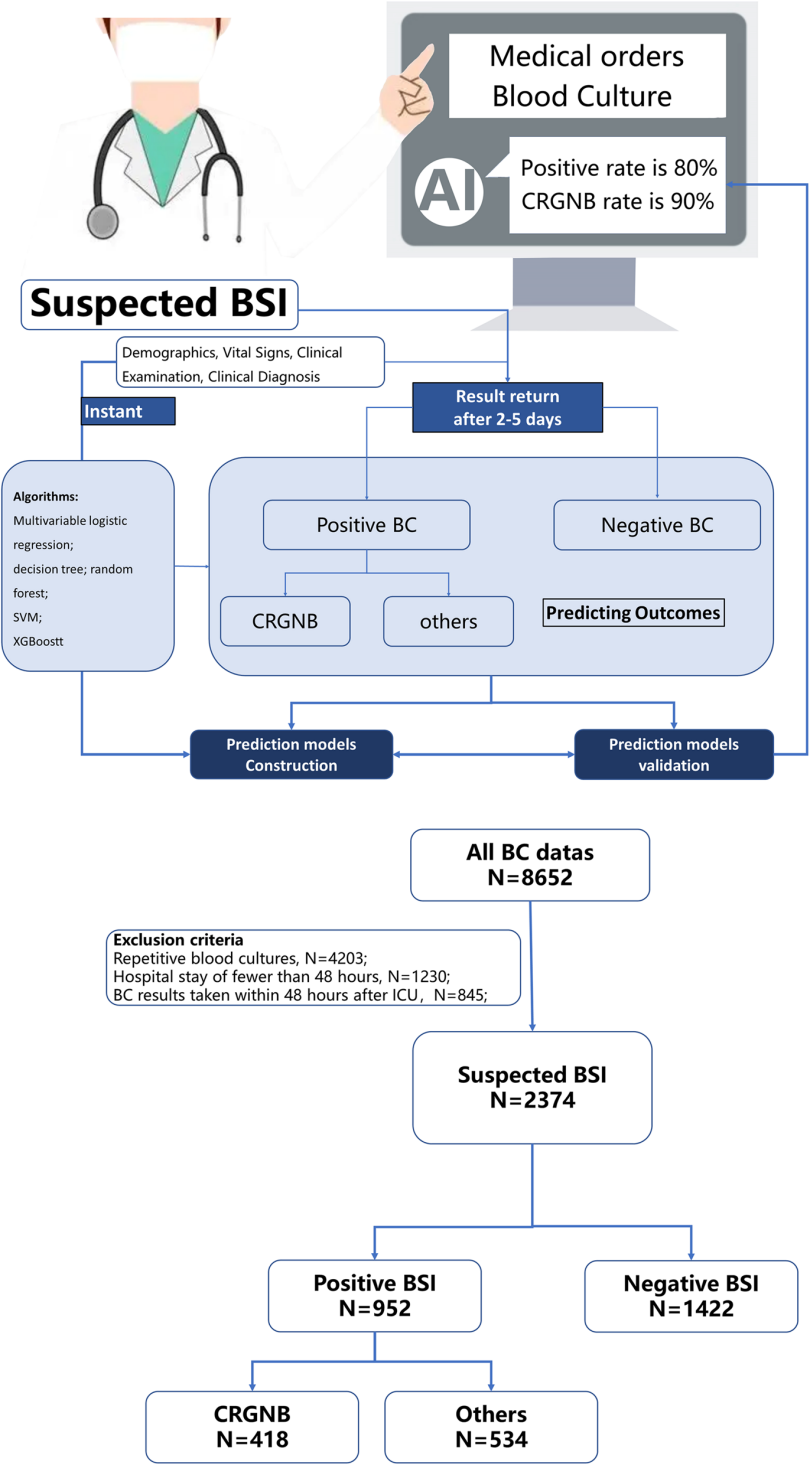
The logical flow of model prediction and flow chart of our study. CR-GNB Carbapenem-resistant gram-negative bacteria, BSI Bloodstream infection, BC Blood culture

It was a regional multi-center retrospective study that included all critically ill patients with blood cultures from 2015.01 to 2021.12. In this study, each blood culture is taken as a time point, and the patient data before the time point are collected to form a complete data. The patients included in the study collected information consisting of demography, diagnosis and complications, vital signs, and laboratory indicators within one day before blood culture sampling. The antibiotic use records and clinical status data were collected two weeks before sampling. Any positive blood culture was marked as a positive patient during hospitalization, but removed the repetitive data with multiple blood culture and only remained the first positive data regardless of whether the subsequent results are positive or not. Only the first blood culture data entry was left in patients with repeated negative blood culture. This study divided patients with suspected bloodstream infections into culture-positive and negative groups. According to the positive results, patients were divided into the CRGNB group and other groups. The machine learning algorithm was used to predict whether the blood culture was positive and whether the pathogen was CRGNB. Figure 1 shows the flow chart. This study has passed the ethics approval of second affiliated Hospital of Zhejiang University school of medicine, and the approval number is IRB-2016–1511.

### Variable definition

Inclusion and exclusion criteria: all patients admitted to the ICU were included in our study. Exclusion criteria: patients with a hospital stay of fewer than 48 h, Blood culture results taken within 48 h after admission into ICU. We retained the first positive blood culture regardless of whether the subsequent results are positive or not and removed others repeatedly. The history of all antibiotic use refers to the records of antibiotics used within two weeks; all laboratory data are taken from the past 24 h of blood culture sampling. If there are duplicate data, take the average. Invasive catheterization defined as the catheterization of invading blood vessels, including central venous catheter, dialysis catheter, catheter of extracorporeal membrane oxygenation, Swan-Ganz, and Pulse index Continuous Cardiac Output. The carbapenems used in our center includes meropenem, imipenem-cilastatin sodium hydrate, ertapenem, biapenem.

### Bacterial detection

Determining and interpreting the minimum inhibitory concentration (MIC) were consistent with the Clinical and Laboratory Standards Institute (CLSI) standards [12, 13]. Carbapenem resistance was defined as reduced susceptibility with a minimum inhibitory concentration of ≥ 2 mg/L for imipenem or meropenem. The definition of tigecycline MIC follows the criteria of the European Committee on Antimicrobial Susceptibility Testing (EUCAST), MIC > 2 mg/L [14]. A laboratory physician conducted the drug susceptibility test with analysis instruments (VITEK2 AST-GN16 France). The broth dilution method determined the strains with mediated sensitivity to tigecycline. We use the blood culture instrument and media system Provided by bioMerieux BacT/ALERT® 3D with Mycobacteria Indication.

### Statistical methods

We use R v.3.6.3 (R Core Team, 2020) and RStudio v.1.4.1029 (RStudio Team, 2020) with related packages to accomplish our data analysis [15]. We use median and quartile spacing presentation for numerical variables with non-normal distribution and use mean and standard deviation for normal distribution data. Extreme values and outliers will be deleted. Then, variables with missing values exceeding 40% were excluded, and cases with missing variables exceeding 50% were removed. Variables with missing values ranging from 10 to 40% were addressed through multiple imputations, while those with less than 10% missing data were filled via simple interpolation [16, 17]. Sensitivity analysis is used to evaluate the stability of multiple interpolation. Multivariate logistic regression and three machine learning algorithms, decision tree, random forest, SVM, and XGBoost, were selected to establish models with corresponding R packages such as "glm," "rpart," "randomForest," "xgboost," and "rattle" [18, 19]. All samples were grouped into a 70% training set, a 15% validation set, and a 15% test set, which was is a relatively common proportion of distribution [8, 20, 21]. The decision tree model produces different branches by calculating the characteristics of independent variables and divides the data into subsets with similar features to achieve classification [19]. Other algorithms are all classification algorithms based on the decision tree. Random forest is an independent but comprehensive decision of hundreds of decision trees with higher accuracy than the decision tree. XGBoost, an improved version of the Gradient Boosting algorithm, builds numerous interrelated decision trees and is highly efficient and flexible [18]. Group comparisons were assessed using an independent sample T-test, a chi-square test, and multivariate logistic regression. Multivariate logistic regression used the step-by-step decreasing method to adjust the parameters to reduce the variable collinearity. The odds ratio (OR) and 95% confidence interval (CI) were calculated to evaluate the association strength. Statistical significance was assigned to a P value of less than 0.05. The evaluation parameters included sensitivity, specificity, positive predictive value, negative predictive value, and AUROC curves. We evaluate the bias of the prediction model through PROBAST framework and write the prediction model through the TRIPOD scheme to ensure the structural integrity of the research [22, 23].

## Results

Approximately 18,000 patients were included in the SHZJU-ICU database, of which 8652 effective blood cultures, and 2375 patients were considered as bloodstream infection during hospitalization. According to the exclusion criteria, 952 patients with positive blood cultures and 1422 negative patients were included (Fig. 1).

**Fig. 2:**
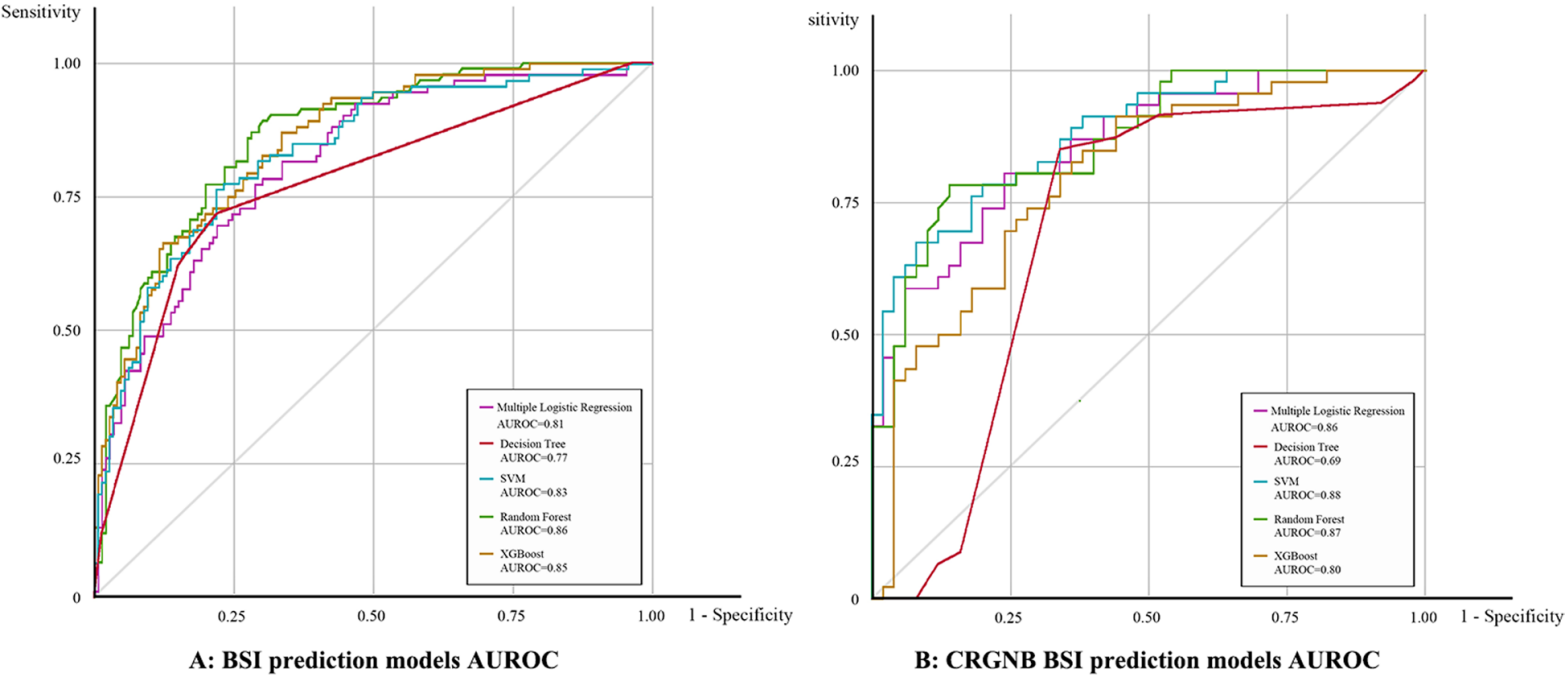
AUROC of the test set for four machine learning algorithm and multivariable logistic regression model in bloodstream prediction model and CRGNB bloodstream prediction model

There was no statistical difference in gender and age between the two groups. The incidence of bloodstream infection was higher in patients with infectious diseases (19.9% vs. 7.4%, *P* < 0.001, OR = 3.10) and internal diseases (6.2% vs. 2.7%, *P* < 0.001, OR = 2.41), and there was no significant difference in other diseases, including trauma, cerebrovascular accident, heart disease, and surgical disease. The positive rate of blood culture was higher in patients with multiple organ dysfunction syndrome, including acute kidney injury (26.1% vs. 16.0%, *P* < 0.001, OR = 1.85) and respiratory failure with mechanical ventilation (72.1% vs. 57.5%, *P* < 0.001, OR = 1.91). Immunosuppressant use (48.8% vs. 28.1%, *P* < 0.001, OR = 2.44) and a history of multiple antibiotics are also high-risk factors for bloodstream infection. All laboratory markers before blood culture are significant and specific high-risk factors can be found in Table 1. The hospitalization time and the stay in the ICU of patients with bloodstream infections were more prolonged than that of negative patients. Meanwhile, the mortality was higher. Multivariate analysis showed that the heart rate (*P* < 0.001, OR = 1.98), temperature (*P* < 0.001, OR = 1.92), procalcitonin (*P* < 0.001, OR = 1.97), and lactic acid (*P* = 0.013, OR = 1.22) before blood culture, a carbapenem (*P* < 0.001, OR = 2.89) and glycopeptide use history (*P* < 0.001, OR = 1.98) were the high factors of bloodstream infection (Table 2). The effectiveness of prediction model shows that the random forest model has the best prediction ability, with AUROC being 0.86, followed by the XGBoost prediction model. The best prediction model accuracy is 77.7%, the highest specificity is 83.6%, and the sensitivity is poor, with a maximum of 70.5% (Table 3) (Fig. 2).

**Table 1:** Baseline characteristic and variables of bloodstream infection in ICU

Variables	Culture-positive group; *N* = 952	Culture-negative group; *N* = 1422	P	OR(95%CI)
Age, yr, (IQR)	59 [41–76]	59 [42–75]	0.44	
Heart rate, (IQR)	110 [96–132]	98 [74–120]	< 0.001	
Systolic pressure, mmHg, (IQR)	111 [93–130]	115 [96–135]	< 0.001	
Diastolic pressure, mmHg, (IQR)	60 [50–76]	60 [46–74]	0.09	
Mean arterial pressure, mmHg, (IQR)	77 [60–94]	79 [62–92]	0.002	
Respiratory rate, times, (IQR)	23 [15-26]	22 [18-28]	< 0.001	
Gender (male)	632 (66.4%)	939 (66.0%)	0.859	1.01 (0.91–1.12)
Fever	438 (46.0%)	439 (30.9%)	< 0.001	1.90 (1.61–2.26)
Primary disease (%)				
Infections	189 (19.9%)	105 (7.4%)	< 0.001	3.10 (2.41–4.01)
Trauma	204 (21.4%)	351 (24.7%)	0.066	1.12 (0.99–1.26)
Cardio-cerebrovascular accident	129 (13.6%)	208 (14.6%)	0.46	1.05 (0.91–1.22)
Heart disease	42 (4.4%)	62 (4.4%)	0.952	0.99 (0.78–1.26)
Postoperative diseases	55 (5.8%)	118 (8.3%)	0.021	1.28 (1.02–1.60)
Internal medicine diseases	59 (6.2%)	38 (2.7%)	< 0.001	2.41 (1.58–3.65)
Malignant tumor	115 (12.1%)	145 (10.2%)	0.15	1.21 (0.93–1.57)
Clinical characteristics (%)				
Leukocytosis	663 (69.6%)	834 (58.6%)	< 0.001	1.61 (1.35–1.92)
Thrombopenia	168 (17.6%)	96 (6.8%)	< 0.001	2.96 (2.26–3.86)
Metabolic acidosis	73 (7.7%)	30 (2.1%)	< 0.001	2.32 (1.56–3.54)
Mechanical ventilation	686 (72.1%)	817 (57.5%)	< 0.001	1.91 (1.60–2.28)
Acute kidney injury	248 (26.1%)	228 (16.0%)	< 0.001	1.85 (1.51–2.26)
Septic shock	280 (29.4%)	335 (23.6%)	0.001	1.35 (1.12–1.63)
History of glucocorticoids	400 (42.0%)	284 (20.0%)	< 0.001	2.90 (2.42–3.48)
Immunosuppression	465 (48.8%)	400 (28.1%)	< 0.001	2.44 (2.06–2.90)
Emergency admission	583 (61.2%)	778 (54.7%)	0.002	1.30 (1.11–1.55)
Invasive catheterization	616 (64.7%)	731 (51.4%)	< 0.001	1.73 (1.46–2.05)
Operation history	516 (54.2%)	675 (47.5%)	< 0.001	0.338 (0.28–0.40)
History of quinolones	81 (8.5%)	43 (3.0%)	< 0.001	2.98 (2.04–4.36)
History of carbapenems	479 (50.3%)	221 (15.5%)	< 0.001	5.50 (4.54–6.67)
History of cephalosporins	454 (47.7%)	456 (32.1%)	< 0.001	1.93 (1.63–2.29)
Antibiotic regimens (%)				
Carbapenems	274 (28.8%)	326 (22.9%)	0.001	1.35 (1.13–1.64)
Tigecycline	200 (21.0%)	69 (4.9%)	< 0.001	5.21 (3.91–6.95)
Colistin or polymyxin	87 (9.1%)	23 (1.6%)	< 0.001	6.12 (3.83–9.76)
Death (%)	223 (23.4%)	98 (6.9%)	< 0.001	1.95 (1.78–2.15)
LOS of infection, days, (IQR)	6.0 [1-26]	5.0 [1-20]	< 0.001	
Length of ICU stay, days, (IQR)	14.7 [3.0–41.3]	9.0 [2.7–28.0]	< 0.001

**Table 2:** Parameters in the multivariable logistic regression model of bloodstream infection in ICU

Variables	P	OR (95%CI)
Heart rate	< 0.001	1.988 (1.783–2.394)
Temperature	< 0.001	1.918 (1.733–2.214)
Lymphocyte count	0.049	1.25 (1.001–1.562)
Procalcitonin	< 0.001	1.976 (1.269–2.684)
Lactic acid	0.013	1.226 (1.071–1.584)
Total bilirubin	0.007	1.596 (1.293–1.899)
Albumin	< 0.001	1.057 (1.037–1.078)
Metabolic acidosis	0.036	1.434 (1.043–1.745)
History of carbapenems	< 0.001	2.899 (2.271–3.702)
History of glucocorticoids	< 0.001	2.253 (1.657–3.061)

**Table 3:** Test set evaluation result of machine learning and multiple logistic regression model for bloodstream infection and CRGNB bacteremia

Model	AUROC	Accuracy	Sensitivity	Specificity	PPV	NPV	F1
Bloodstream infection model							
Logistic regression	0.81	74.3	65.2	80.1	67.4	78.5	0.66
Decision Tree	0.77	76.0	64.1	83.6	71.1	78.7	0.67
Random Forest	0.86	77.7	69.6	82.9	71.9	81.2	0.71
SVM	0.83	76.5	66.3	82.8	70.9	79.6	0.69
XGBoost	0.85	76.4	70.6	80.1	69.1	81.2	0.69
CRGNB bacteremia model							
Logistic regression	0.86	74.0	87.0	62.0	67.8	83.8	0.76
Decision Tree	0.69	70.8	86.9	56.0	64.5	82.3	0.74
Random Forest	0.87	70.8	89.1	54.0	64.0	84.4	0.75
SVM	0.88	75.0	87.0	64.0	69.0	84.2	0.77
XGBoost	0.80	70.8	79.5	58.0	65.0	80.5	0.73

*PPV* Positive likelihood ratio, *NPV* Negative predictive value, *AUROC* Area under the receiver operating characteristic

**Fig. 3:**
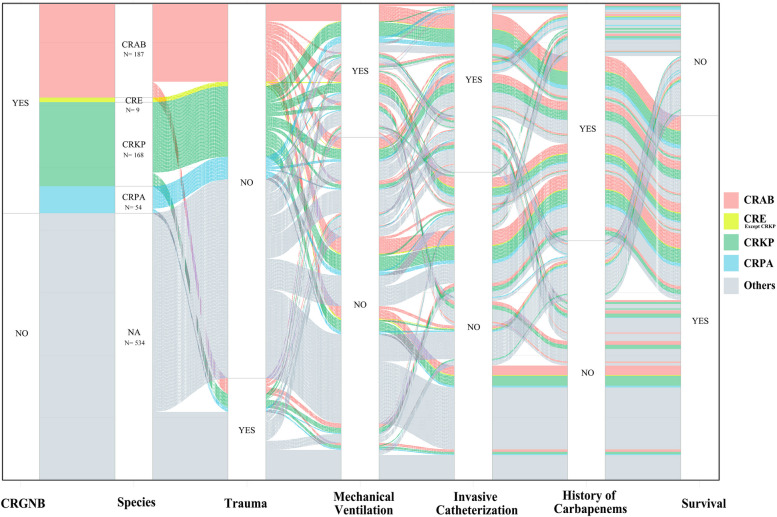
The relationship between different species of CRGNB and high-risk factors including mechanical ventilation, invasive catheterization, and carbapenem use history

Among patients with positive blood cultures, there were 418 patients in the CRGNB group and 534 in the non-CRGNB group. Among the 418 cases of CRGNB bloodstream infection, CRKP and CRAB accounted for 168 and 187 cases respectively, with CRPA of 54 cases, CRE of 9 cases (supplement Table 1). The incidence of CRGNB bloodstream infection in trauma patients was higher, and there was no statistical difference in other diseases. Mechanical ventilation, history of glucocorticoid use, immunosuppressive condition, invasive catheterization, quinolone, carbapenem, and cephalosporin use history are high-risk factors for CRGNB bloodstream infection (Table 4). In multivariate analysis, the main high-risk factors were mechanical ventilation, invasive catheterization, and carbapenem use history (Table 5). Sankey Diagram can visually show the relationship between different species of CRGNB and high-risk factors in Fig. 3.

**Table 4:** Baseline characteristic and variables of CRGNB Bacteremia in ICU

Variables	CRGNB *N* = 418	Non-CRGNB *N* = 534	P	OR(95%CI)
Age, yr, (IQR)	60 [43–78]	60 [43–77]	0.76	
Heart rate, (IQR)	112 [88–132]	109 [87–130]	0.075	
Systolic pressure, mmHg, (IQR)	111 [87–135]	111 [89–135]	0.771	
Diastolic pressure, mmHg, (IQR)	60 [45–77]	60 [45–73]	0.795	
Mean arterial pressure, mmHg, (IQR)	77 [59–94]	76 [60–94]	0.769	
Respiratory rate, times, (IQR)	24 [18-31]	23 [18-28]	0.028	
Gender (male)	258 (61.7%)	374 (70.1%)	0.007	0.69 (0.53–0.90)
Fever	209 (50.0%)	229 (42.9%)	0.03	1.33 (1.03–1.72)
Primary disease (%)				
Infections	91 (21.7%)	98 (18.3%)	0.18	1.23 (0.90–1.70)
Trauma	108 (25.8%)	96 (17.9%)	0.003	1.59 (1.16–2.17)
Cardio-cerebrovascular accident	59 (14.1%)	70 (13.1%)	0.653	1.09 (0.75–1.58)
Heart disease	21 (5.0%)	21 (3.9%)	0.416	1.29 (0.69–2.39)
Postoperative diseases	25 (6.0%)	30 (5.6%)	0.810	1.07 (0.62–1.85)
Internal medicine diseases	26 (6.2%)	33 (6.1%)	0.980	1.007 (0.59–1.72)
Malignant tumor	51 (12.2%)	64 (11.9%)	0.919	1.021 (0.68–1.51)
Clinical characteristics (%)				
Leukocytosis	291 (69.6%)	372 (69.7%)	0.988	0.998 (0.75–1.31)
Thrombopenia	89 (21.2%)	79 (14.8%)	0.09	1.55 (1.11–2.18)
Metabolic acidosis	42 (10.0%)	31 (5.8%)	0.015	1.81 (1.12–2.94)
Mechanical ventilation	349 (83.5%)	337 (63.1%)	< 0.001	2.95 (2.16–4.04)
Acute kidney injury	114 (27.2%)	134 (25.1%)	0.447	1.11 (0.84–1.49)
Septic shock	136 (32.5%)	144 (26.9%)	0.061	1.31 (0.98–1.73)
History of glucocorticoids	213 (50.9%)	187 (35.0%)	< 0.001	1.92 (1.42–2.50)
Immunosuppression	245 (58.6%)	220 (41.2%)	< 0.001	2.02 (1.55–2.62)
Emergency admission	244 (58.3%)	339 (63.4%)	0.108	0.81 (0.62–1.05)
Invasive catheterization	312 (74.6%)	304 (56.9%)	< 0.001	2.23 (1.69–2.94)
Operation history	236 (56.4%)	280 (52.4%)	0.216	1.17 (0.91–1.52)
History of quinolones	51 (12.2%)	30 (5.62%)	< 0.001	2.34(1.45–3.74)
History of carbapenems	279 (66.7%)	200 (37.5%)	< 0.001	3.35 (2.56–4.38)
History of cephalosporins	232 (55.5%)	222 (41.6%)	< 0.001	1.75 (1.35–2.27)

There are 15 variables are included in Supplemental Table 1 due to the limited space

*IQR* Interquartile range

**Table 5:** Parameters in the multivariable logistic regression model of CRGNB Bacteremia in ICU

Variables	P	OR (95%CI)
C-reactive protein	0.025	1.28 (1.06–1.59)
prothrombin time	0.011	1.34 (1.11–1.48)
Trauma	0.002	1.78 (1.22–2.61)
Mechanical ventilation	< 0.001	1.94 (1.35–2.81)
Invasive catheterization	< 0.001	1.66 (1.22–2.27)
History of carbapenems	< 0.001	2.84 (2.05–3.94)

In the CRGNB prediction model analysis, the SVM and random forest model have higher AUROC curves, which are 0.88 and 0.87, respectively (Table 3). In the model performance, the sensitivity is significantly improved compared to the blood flow infection prediction model, and the sensitivity of random forest model is 89.1. The SVM model has the highest overall accuracy, reaching 75%.

## Discussion

This study mainly presents the following clinical scenario: when clinicians suspect bloodstream infection and make blood culture orders, artificial intelligence predicts the positive rate of blood culture and the probability caused by CRGNB culture result based on previous data. There are some clinical studies on the predictive model of positive blood culture, but no research of CRGNB. This study shows that the artificial intelligence algorithm has the potential to predict the occurrence of nosocomial CRGNB bloodstream infection accurately. Limited by the longer blood culture cycle and fewer choices of antibiotics, it is necessary to predict CRGNB bloodstream infection earlier so that the empirical antimicrobial regimen can be transformed into the target antimicrobial regimen more quickly and the use of unnecessary broad-spectrum antibiotics can be reduced [24].

In this study, the AUROC of our bloodstream infection prediction model was 0.86. It was similar to the previously published bloodstream infection prediction model, and the AUROC was between 0.82 and 0.926 [6–10]. The judgment of the outcome of the bloodstream infection prediction model is based on the results of blood culture, and this outcome index is very consistent with the construction of the binary machine learning algorithm which is better than traditional multivariate logistic model based on multi-dimensional data analysis. But the same problem with these studies is that blood culture is not reliable. Blood culture can be regarded as bloodstream infection, but bloodstream infection is not necessarily positive blood culture. Before blood culture breaks through its own defects, false negative is almost inevitable. Although there are many blood culture results in our study, we only choose the results of the first sampling in patients. Follow-up blood culture may lead to misleading due to the use of various antibiotics. In addition, continuous negative results may lead to a significant increase in negative samples, resulting in inadequate model fitting. These factors are often ignored in other studies, resulting in false high accuracy.

On this basis, the accuracy of predicting bloodstream infection caused by CRGNB was excellent, with an AUROC of 0.88. There are very few studies on this perspective. The researchers conducted a retrospective study of patients with hematologic diseases in a tertiary hospital in Barcelona. Typically, 3235 episodes of neutropenia and 180 infections (5.6%) were recorded in 349 patients. The machine learning algorithm predicted the incidence of MDR-GNB infection with an AUROC of 0.79 [25]. Our research has a more significant amount of data and a precise prediction result, which is of great significance in improving the accuracy of the prediction model.

For ICU patients suspected of bloodstream infection, the positive rate of blood culture in patients with severe sepsis before the use of antibiotics was 31.4% to 50.6% [26, 27]. The positive rate of blood culture was approximately 40%. A prospective study of patients in medical wards of 31 centers in Italy found that the incidence of MDR-GNB bloodstream infection was 48.2%. High-risk factors included advanced age, previous hospitalization history, and history of antibiotic use [28]. It seems to confirm the current epidemic trend of drug-resistant bacteria that CRGNB accounts for nearly half of the patients with positive blood culture in our central, similar to the international epidemiological data. The isolation rate of CRGNB in hospital-acquired infections is generally high. Statistics worldwide show that the isolation rate of CRGNB in Southeast Asia varies from 26 to 65% [27]. Our data do not fully represent the epidemiological characteristic of CRGNB bloodstream infection in our center, which is related to the exclusion criteria of this study. We removed bloodstream infections from community sources as much as possible. At the same time, it is difficult to achieve no antibiotic exposure if bloodstream infection occurs during hospitalization in the ICU, which reduces the detection rate of sensitive bacteria and adds to the detection rate of MDR in the study. Therefore, the data of our center only show ICU-related CRGNB bloodstream infection to a certain extent. There were KPC-2 in 96.7% genotype of the CRKP through epidemic survey in Zhejiang, China. We did not carry out genotype testing for CRPA and CRAB, so we did not present genotype in the article [29–31]. Our study provides a reference for nosocomial prevention and control, identifies patients with high-risk drug-resistant bacteria infection and implements more accurate contact isolation and hand hygiene to reduce costs and increase efficiency [32]. The prediction model provides some preliminary references for antibiotic management. With the increase of the amount of data and model optimization, the prediction model can provide more convincing guidance and suggestions.

The accuracy of the bloodstream infection prediction model and AUROC is not very high in our research. This may be related to the low positive rate of blood culture, and there are many false negative cases and false positive due to contaminations. Due to varying sampling times and early antibiotic exposure, many septic shock patients were considered as bloodstream infections but with negative blood cultures. These patients are classified as negative cases, increasing the uncertainty of predictive model classification [33]. There are false positive and false negative, which is related to the level of hospitals, the timing of blood culture and the history of the use of antibiotics. Although there are next generation sequencing (NGS) and polymerase chain reaction (PCR) results as a supplement [34], the status of blood culture as a gold standard cannot be shaken in the short term. Whether we can include PCR or NGS results to enhance the diagnosis of bloodstream infection needs more literature support. With the continuous improvement of detection technology, the diagnosis of bloodstream infection is more accurate, and the prediction model will be more accurate. This study is a retrospective study of a single database; as a regional multi-center database, the data source is relatively simple, and the prediction model may have the risk of poor external adaptability. There are errors, biases and deficiencies in the clinical variables in the retrospective study. In particular, continuous variables, such as the recording of heart rate, temperature and blood pressure may lead to inaccurate data interpretation due to errors in analytical methods, which may affect the accuracy of model construction. It was a similar limitation in all research by the MIMIC database. Currently, the overall accuracy of artificial intelligence prediction model of CRGNB bloodstream infection is not very high, and the distance to guide the use of clinical antibiotics requires more scenario application practice, which must be confirmed by prospective and even randomized controlled trial research. At the same time, our model also has the problem of external validation, because the incidence of bloodstream infection is different in different MDR epidemic areas. Low and high epidemic areas of CRGNB may not be able to share the same model, which can only be achieved by adjusting the parameters or even modifying the model framework or even reconstructing a model.

## Conclusion

Machine learning algorithm can accurately predict the occurrence of ICU-acquired bloodstream infection and identify whether CRGNB causes it. This can provide more references for clinicians to make antibiotic decisions.

### Supplementary Information

**Supplementary Material 1.**

**Supplementary Material 2.**

**Supplementary Material 3.**

## Data Availability

The datasets used and/or analysed during the current study are available from the corresponding author on reasonable request.

## Abbreviations

CRGNB: Carbapenem-Resistant Gram-Negative Bacterial
MDR: Multiple Drug-Resistant Bacteria
CRE: *Carbapenem* *-Resistant* *Enterobacteriaceae*
CRANB: Carbapenem-Resistant *Acinetobacter **Baumannii*
CRPA: Carbapenem-resistant *Pseudomonas aeruginosa*
CRKP: Carbapenem-resistant *Klebsiella** Pneumoniae*
ICU: Intensive Care Unit
MIC: Minimum Inhibitory Concentration
CLSI: Clinical and Laboratory Standards Institute
OR: Odds Ratio
CI: Confidence Interval
AUROC: Area Under the Receiver Operating Characteristic
NGS: Next Generation Sequencing
PCR: Polymerase Chain Reaction
