# Supplementary material for: Prediction of carbapenem-resistant gram-negative bacterial bloodstream infection in intensive care unit based on machine learning
Source: BMC Med Inform Decis Mak. 2024 May 14;24:123. doi: 10.1186/s12911-024-02504-4 (PMC11095031; doi:10.1186/s12911-024-02504-4)
Supplement: Supplementary file 1 — Supplementary Material 1. [file 12911_2024_2504_MOESM1_ESM.docx]

Supplement Table 1: The distribution of bacteria isolated from the 952 cases of bloodstream infection

| >Species | Resistance to carbapenem | Susceptible |
| --- | --- | --- |
| *Klebsiella pneumoniae* | **168** | **111** |
| *Pseudomonas aeruginosa* | **54** | **76** |
| *Acinetobacter baumannii* | **187** | **34** |
| *Escherichia coli* | **4** | **71** |
| *Enterobacter cloacae* | **2** | **34** |
| *Citrobacter freundii* | **1** | **9** |
| Another gram-negative bacterium | **2** | **5** |
| *Staphylococcus* | **87** | **25** |
| *Enterococcus* | **0** | **30** |
| Another gram-positve bacterium | **34** | **18** |
